# Prognosis Prediction Through an Integrated Analysis of Single-Cell and Bulk RNA-Sequencing Data in Triple-Negative Breast Cancer

**DOI:** 10.3389/fgene.2022.928175

**Published:** 2022-07-01

**Authors:** Xiangru Wang, Hanghang Chen

**Affiliations:** ^1^ Department of General Surgery, The Affiliated Hospital Of Henan Medical College, Henan Medical College Hospital Workers, Zhengzhou, China; ^2^ Southern Medical University, Guangzhou, China

**Keywords:** Triple-negative breast cancer (TNBC), prognosis, single cell, immune infiltration, tumor mutational burden

## Abstract

**Background:** Genomic and antigenic heterogeneity pose challenges in the precise assessment of outcomes of triple-negative breast cancer (TNBC) patients. Thus, this study was designed to investigate the cardinal genes related to cell differentiation and tumor malignant grade to advance the prognosis prediction in TNBC patients through an integrated analysis of single-cell and bulk RNA-sequencing (RNA-seq) data.

**Methods:** We collected RNA-seq and microarray data of TNBC from two public datasets. Using single-cell pseudotime analysis, differentially expressed genes (DEGs) among trajectories from 1534 cells of 6 TNBC patients were identified as the potential genes crucial for cell differentiation. Furthermore, the grade- and tumor mutational burden (TMB)-related DEGs were explored via a weighted correlation network analysis using the Molecular Taxonomy of Breast Cancer International Consortium dataset. Subsequently, we utilized the DEGs to construct a prognostic signature, which was validated using another independent dataset. Moreover, as gene set variation analysis indicated the differences in immune-related pathways between different risk groups, we explored the immune differences between the two groups.

**Results:** A signature including 10 genes related to grade and TMB was developed to assess the outcomes of TNBC patients, and its prognostic efficacy was prominent in two cohorts. The low-risk group generally harbored lower immune infiltration compared to the high-risk group.

**Conclusion:** Cell differentiation and grade- and TMB-related DEGs were identified using single-cell and bulk RNA-seq data. A 10-gene signature for prognosis prediction in TNBC patients was constructed, and its performance was excellent. Interestingly, the signature was found to be closely related to tumor immune infiltration, which might provide evidence for the crucial roles of immune cells in malignant initiation and progression in TNBC.

## Introduction

Triple-negative breast cancer (TNBC) is defined as the absence of estrogen receptor (ER) and progesterone receptor (PR) expression and human epidermal growth factor receptor (HER2) amplification. TNBC patients could not benefit from endocrine or anti-HER2 therapies, and chemotherapy is currently one of the few proven therapeutic choices. Considering the unfavorable prognosis and aggressive clinicopathological characteristics of TNBC, new treatments are warranted and clinical therapeutic options should be individualized to optimize benefits.

Traditional molecular subtypes based on ER, PR, and HER-2 expression could partly explain the distinction; however, they might have limitations for patient-tailored treatment strategies (Yersal and Barutca, 2014). In this regard, the microarray (Abd-Elnaby et al., 2021) and next-generation sequencing (NGS) (Hong et al., 2020) technologies with high accuracy and reduced cost might be optimal choices in cancer screening and treatment. Furthermore, individual gene expression analysis could provide new chances for individualized cancer prognosis prediction in addition to clinicopathological features (Lal et al., 2017). The question now, of course, is how we translate the RNA-sequencing (RNA-seq) data into clinical application.

Another problem is that RNA-seq of the bulk tissue only indicates an average gene expression of all cells in a tumor microenvironment (TME). The advent of single-cell RNA sequencing (scRNA-seq), first issued in 2009 (Tang et al., 2009), has facilitated our understanding of the TME at an individual cell level and in terms of cell–cell interplay (Chen et al., 2019). In recent years, rapid progress in the development of scRNA-seq has provided insights into the heterogeneity of hundreds of thousands of cells in multiple tumors (Chung et al., 2017; Zheng et al., 2018; Fu et al., 2020). By bridging the gap between single-cell and bulk RNA-seq (Malikic et al., 2019), scRNA-seq will contribute to the illumination of tumorigenesis, thereby advancing the identification of new therapeutic targets.

Hereby, in this study, we identified the differentially expressed genes (DEGs) among trajectories through pseudotime analysis and utilized DEGs to construct a robust prognostic signature through an integrated analysis of single-cell and bulk RNA-seq data. The signature also correlated with immune infiltration, which suggests a potential connection between the crucial genes in tumor malignant grade and immune infiltration of TNBC patients.

## Materials and Methods

### Data Collection

The scRNA-seq data of 1534 cells from 6 TNBC patients were downloaded from the Gene Expression Ominibus (GEO) database (https://www.ncbi.nlm.nih.gov/geo/, GSE118389) (Karaayvaz et al., 2018).

The microarray data of female TNBC patients and clinical information of one validation cohort (including 107 samples) were collected from the GEO database (GSE58812) (Jézéquel et al., 2015).

The microarray data of 299 TNBC patients and the corresponding clinical data were collected from the Molecular Taxonomy of Breast Cancer International Consortium (METABRIC) project in the cBioPortal database (http://www.cbioportal.org/) (Pereira et al., 2016).

### Single-Cell RNA-Sequencing Data Analysis

We used the “Seurat” R package (version 4.0.4) to remove unqualified cells with a threshold (the gene counts per cell >50 and the percentage of mitochondrial genes per cell <5). We normalized the expression of cells using the LogNormalize function and identified 2000 most highly variable genes among the cells.

We conducted principal component analysis (PCA) and calculated the *p*-value of each principal component (PC). We selected 20 as dimensions of reduction and performed the t-distributed stochastic neighbor embedding (t-SNE) analysis. Then, we clustered cells with the identified marker genes (|log fold change (FC)| ≥1 and adjusted the *p*-value ≤0.05). Next, we utilized the “SingleR” (version 1.8.1) to annotate cell types. To validate the accuracy of the cell annotation, we summarized some cell markers, such as CD3D for T cells, and displayed the cell markers in a cell landscape.

We envisaged that some genes were crucial for cell differentiation in the TME and, therefore, conducted pseudotime analysis with the “Monocle” R package (version 2.22) (Qiu et al., 2017). Cells were ordered along different trajectories, which meant a different developmental stage. The DEGs among the trajectories were identified for subsequent analyses.

### Identification of the Grade- and Tumor Mutational Burden-Related Differentially Expressed Genes Through Weighted Gene Coexpression Network Analysis

To explore the genes associated with tumor progression and malignant grade, we conducted weighted gene coexpression network analysis (WGCNA) to pick out grade- and tumor mutational burden (TMB)-related genes from the DEGs (Langfelder and Horvath, 2008). The expression data of DEGs and clinical data of the METABRIC project were imported and analyzed using the “WGCNA” R package (version 1.70-3). We calculated the optimal softPower with the pickSoftThreshold function (Zhang et al., 2018) and classified genes in different modules. We calculated the correlations among gene modules, grade and TMB using Pearson’s correlation analysis and visualized them in a heatmap. We selected the modules that were significantly correlated with grade and TMB for further study.

### Construction and Validation of a Prognostic Signature

We normalized the bulk RNA expression data of the METABRIC and GEO cohorts using the “limma” and “sva” R packages (Yang et al., 2018).

We randomly selected 70% of TNBC patients in the METABRIC cohort as a training cohort. We conducted univariate Cox regression in the “survival” R package to screen out genes related to prognosis in the training cohort. Then, we applied the least absolute shrinkage and selection operator (LASSO) regression in the “glmnet” R package to develop a prognostic signature. The risk score of each patient was calculated as ∑ (expression of gene*i* ∗ β*i*), where *β* denotes the coefficient of every gene in the signature.

We stratified TNBC patients in the training, test, and GEO cohorts separately into low- and high-risk groups based on the median risk score of the training cohort and compared overall survival (OS) using the Kaplan–Meier (KM) method (“survival” R package).

To further validate the efficacy of the signature, we employed the “survminer” and “time-ROC” R packages to perform 3-, 5-, and 8-year receiver operating characteristic curve (ROC) analyses.

To assess the performance of the signature in independent prognosis prediction, we extracted clinical information (age, stage, and grade) and TMB in the METABRIC cohort and conducted univariate and multivariate Cox regression.

### Nomogram Construction and Validation

To improve the efficacy of the signature, we considered significant clinical factors (age and stage) and constructed a nomogram via “rms” R package in the METABRIC cohort.

To validate the efficacy of the nomogram, we drew a calibration plot to visualize the consistency of the predicted and observed OS. We also plotted 3-, 5-, and 8-year ROC curves to assess the efficacy of the nomogram.

We used the concordance index (C-index) to assess the prognostic efficacy of the nomogram compared to clinical factors using the “pec” R package.

### Exploration of the Expression of the Genes in Particular Cells

To determine the expression of the signature genes in particular cells, we further visualized the expression levels of the signature genes in a single-cell atlas.

### Gene Set Variation Analysis

We calculated and compared the different enrichment pathways between different risk groups using the gene set variation analysis (GSVA) R package (Hänzelmann et al., 2013). We visualized the differences in a heatmap.

### Immune Infiltration Differences Between Different Risk Groups

Considering the differences in immune-related pathways through GSVA, we calculated the immune infiltration score of every TNBC sample from the METABRIC dataset. We also compared the differences in the immune cells and pathways between different risk groups.

We calculated the correlations among the risk score, the genes in the signature, and immune cells and pathways. We displayed the correlations using a heatmap.

Given the close correlations above, we also calculated the correlations among the risk score, the genes in the signature, and immune checkpoint genes.

### Statistical Analysis

The Wilcoxon test was utilized to compare the differences between the two groups. Association between variables was determined using the Spearman correlation test. The chi-square test was used to compare the categorical variables. All statistical analyses were performed using the R software (v4.1.0), and *p* < 0.05 was considered statistically significant. Furthermore, we utilized the “set.seed” function to guarantee the reproducibility of the research.

## Results

### Cell Differentiation-Related Genes Obtained Through Analysis of scRNA-Sequencing Data

**FIGURE 1:**
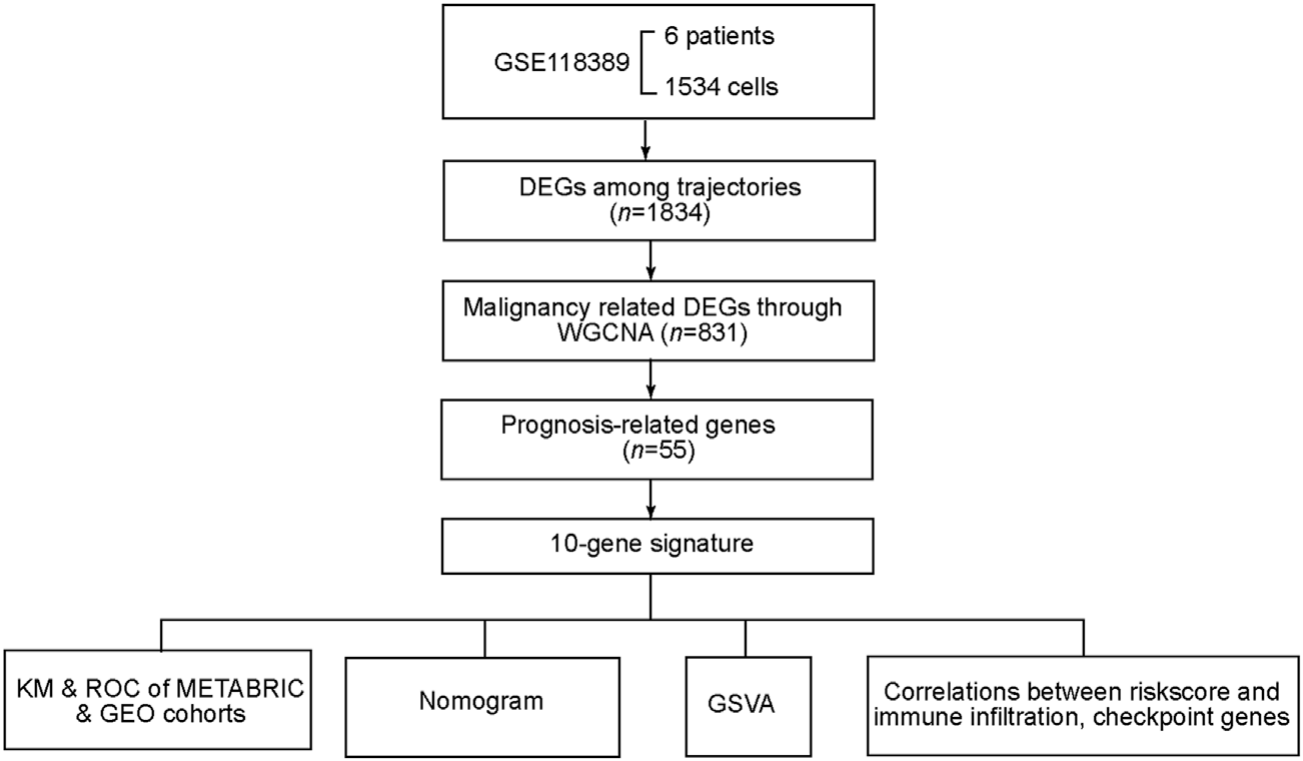
Flowchart of data processing and analyses.

**FIGURE 2:**
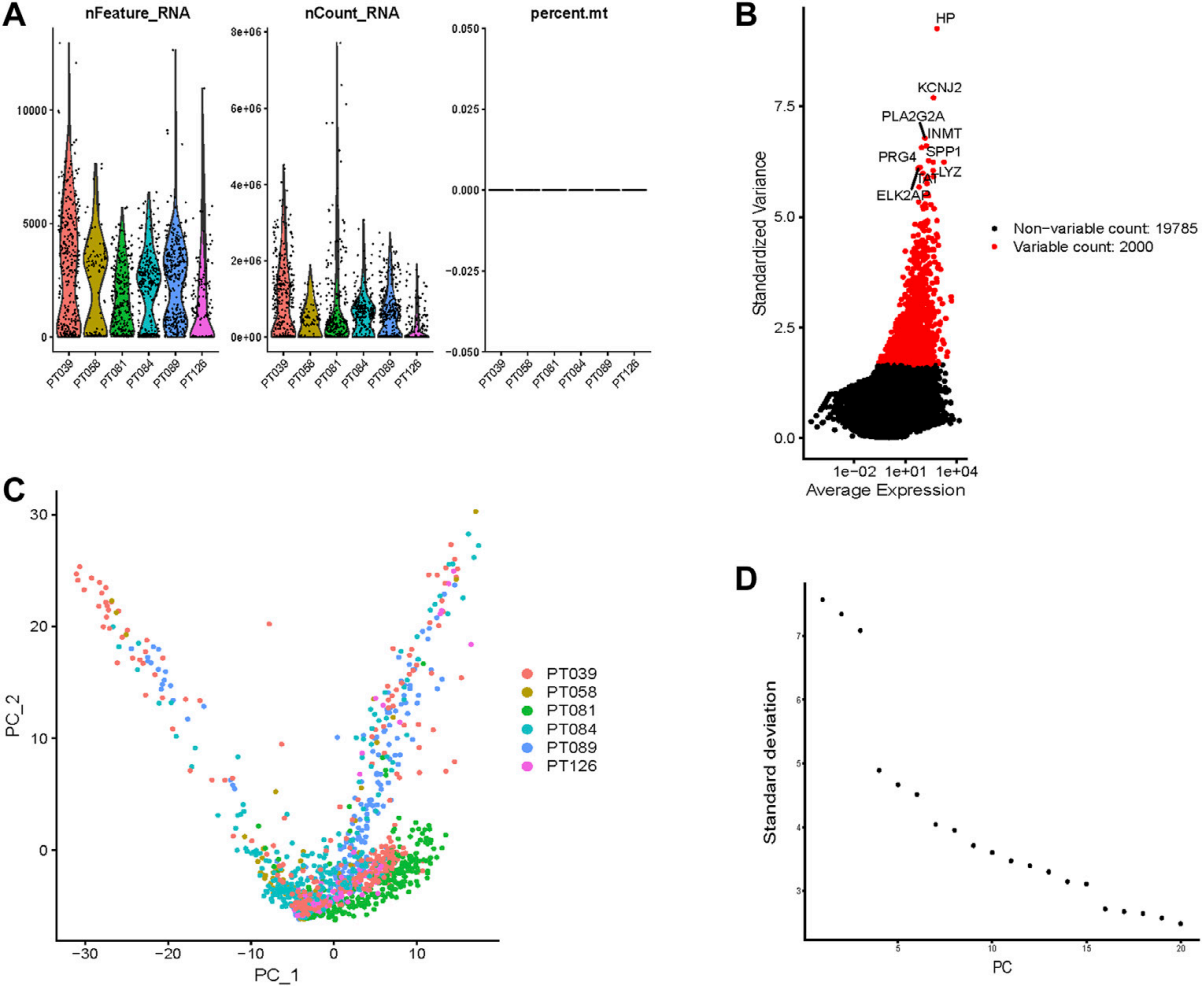
Normalization, filtration, and dimension reduction of single-cell RNA-seq data. **(A)** The gene counts per cell (nFeature_RNA), number of unique molecular identifiers (UMIs) per cell (nCount_RNA), and percentage of mitochondrial genes per cell (percent.mt) of the single-cell RNA-seq data. **(B)** The top 2000 variable DEGs between cells are marked in red color, and the 10 most highly variable DEGs are labeled. **(C)** The top two dimensions of all tumor cells. **(D)** An elbow plot of the standard deviation of each principal component (PC) to help PC selection.

The flowchart of this study is displayed in Figure 1. We selected cells with >50 gene counts per cell (nFeature_RNA) and <5% mitochondrial gene counts (percent.mt, Figure 2A). nFeature_RNA showed a positive correlation with nCount_RNA (number of detected molecules/unique molecular identifiers, Supplementary Figure S1A), which indicated the influence of sequencing depth and rationalized the normalization of data. The top 2000 variable DEGs between cells were marked with red color, and the 10 most highly variable DEGs were labeled (Figure 2B). For the 2000 highly variable genes, we conducted PCA and visualized the top 30 feature genes of the top four PCs (Supplementary Figure S1B). Figure 2C shows the top two dimensions of all tumor cells. We calculated the *p*-values of each PC (Supplementary Figure S1C) and plotted an elbow plot (Figure 2D), which assisted in the selection of 1–20 PCs in the downstream analyses.

**FIGURE 3:**
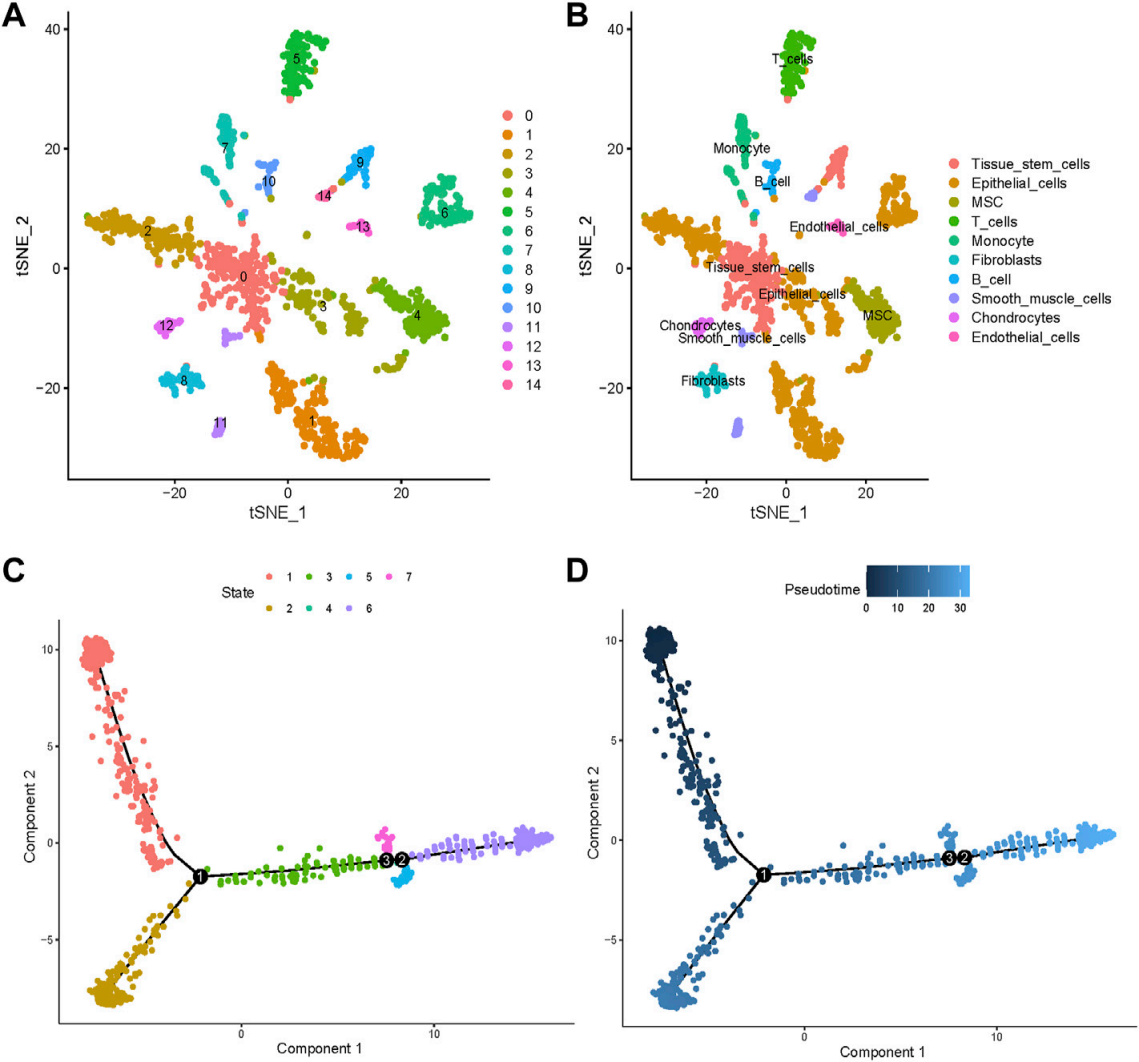
Cell clusters and types of annotation and pseudotime analysis. **(A)** Fourteen different cell clusters were identified by performing t-distributed stochastic neighbor embedding (t-SNE). **(B)** Cell types were further annotated and labeled by exploiting the cell markers. **(C)** All cells were ordered along trajectories to construct a pseudotime axis. Different colors represent different states. **(D)** The deeper the color, the earlier the beginning of cell progressions.

Variable genes helped classify all cells into 15 clusters that were presented through t-SNE (Figure 3A). We annotated 10 cell types and labeled them as shown in Figure 3B. To validate the accuracy of cell annotation, we summarized some marker genes and displayed their expressions in different clusters (Supplementary Figure S2A).

Pseudotime analysis simulated the cell developmental states based on the gene expression. All cells were ordered along trajectories to construct a pseudotime axis (Figures 3C,D). Cell types are annotated in Supplementary Figure S2B and the significant DEGs among the different trajectories that indicated differences in cell differentiation are listed in Supplementary Table S1.

### Identification of Genes Related to Grade and TMB

**FIGURE 4:**
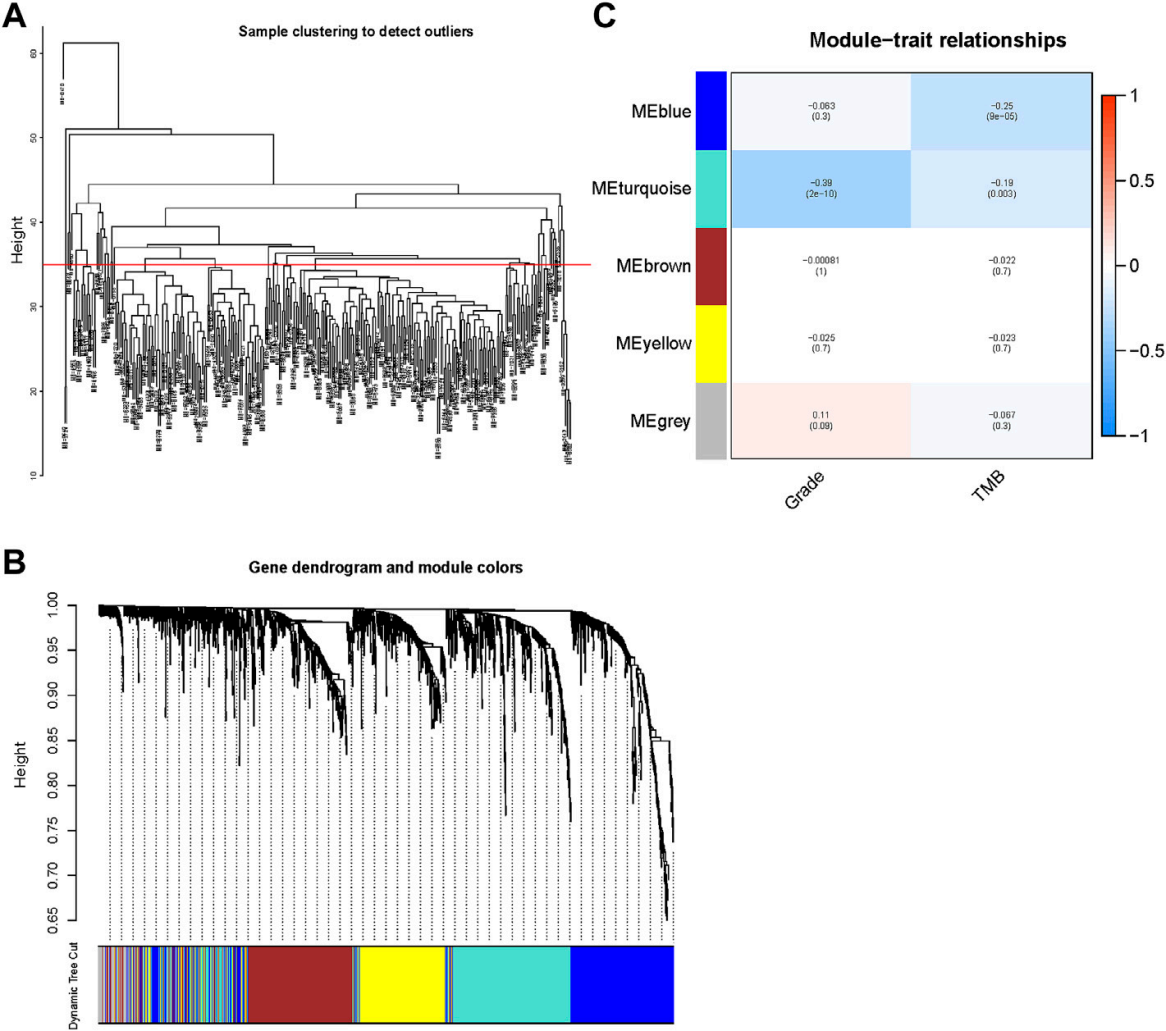
Identification of genes related to the TNBC grade and tumor mutational burden (TMB) through weighted gene coexpression network analysis (WGCNA). **(A)** All samples were clustered and displayed. We selected 35 as the cutHeight. **(B)** A gene dendrogram was generated, and genes were clustered into different modules with different colors. **(C)** The correlations between gene modules and grade and TMB were calculated using Pearson’s correlation and exhibited in a heatmap. Blue and turquoise gene modules containing 831 genes were found to be significantly related to grade and TMB (*p* < 0.05).

All samples from the METABRIC dataset were clustered (Figure 4A). We chose 35 as the cutHeight to remove outliers from all samples. By calculation, 4 was selected as the optimum soft threshold power (Supplementary Figure S3A) to generate five modules (Supplementary Figure S3B). A gene dendrogram was generated, and genes were clustered into different modules with different colors (Figure 4B). The correlations among gene modules, grade, and TMB are displayed in Figure 4C. Among the modules, the blue and turquoise gene modules containing 831 genes were found to be significantly related to grade and TMB, which may correlate with tumor progression and malignant grade. These genes were collected for the downstream analyses.

### Identification and Validation of a 10-Gene Signature

**FIGURE 5:**
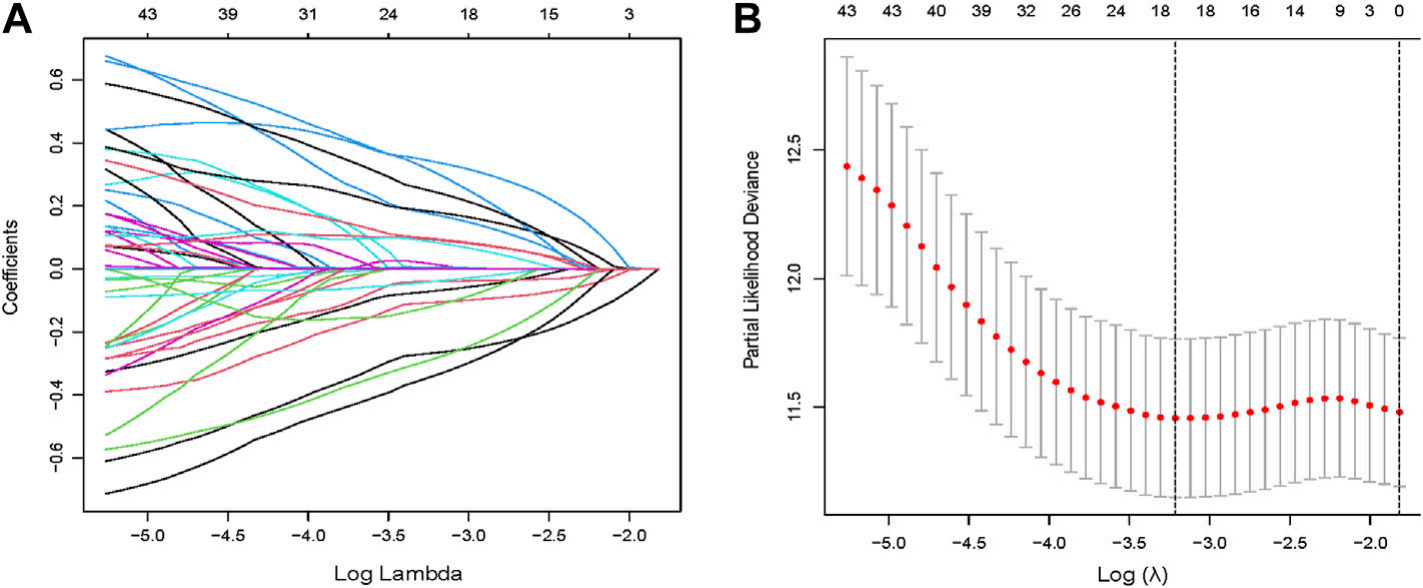
Identification of a prognostic signature in the training cohort. **(A,B)** Through LASSO regression, a signature including 10 genes was developed based on the optimal *λ*.

Through univariate Cox regression, 55 genes were prognostic and are listed in Supplementary Table S2. Through LASSO regression, a signature including 10 genes was developed based on the optimal *λ* (Figures 5A,B). Table 1 lists the 10 genes and their coefficients that could be applied to calculate the risk score of every sample.

**TABLE 1:** Genes involved in the signature and their coefficients.

No.	Gene	Coefficient
1	RMND5A	0.581127231277829
2	ZNF829	0.568292268207948
3	KDM5B	0.391798557667475
4	NCBP2	0.339989521789735
5	GPI	0.250924449438002
6	BGN	0.181013977037171
7	CCND2	−0.262004038399515
8	PLBD1	−0.410074410432122
9	ZYG11A	−0.461328087202662
10	IL17RD	−0.608201023192413

**FIGURE 6:**
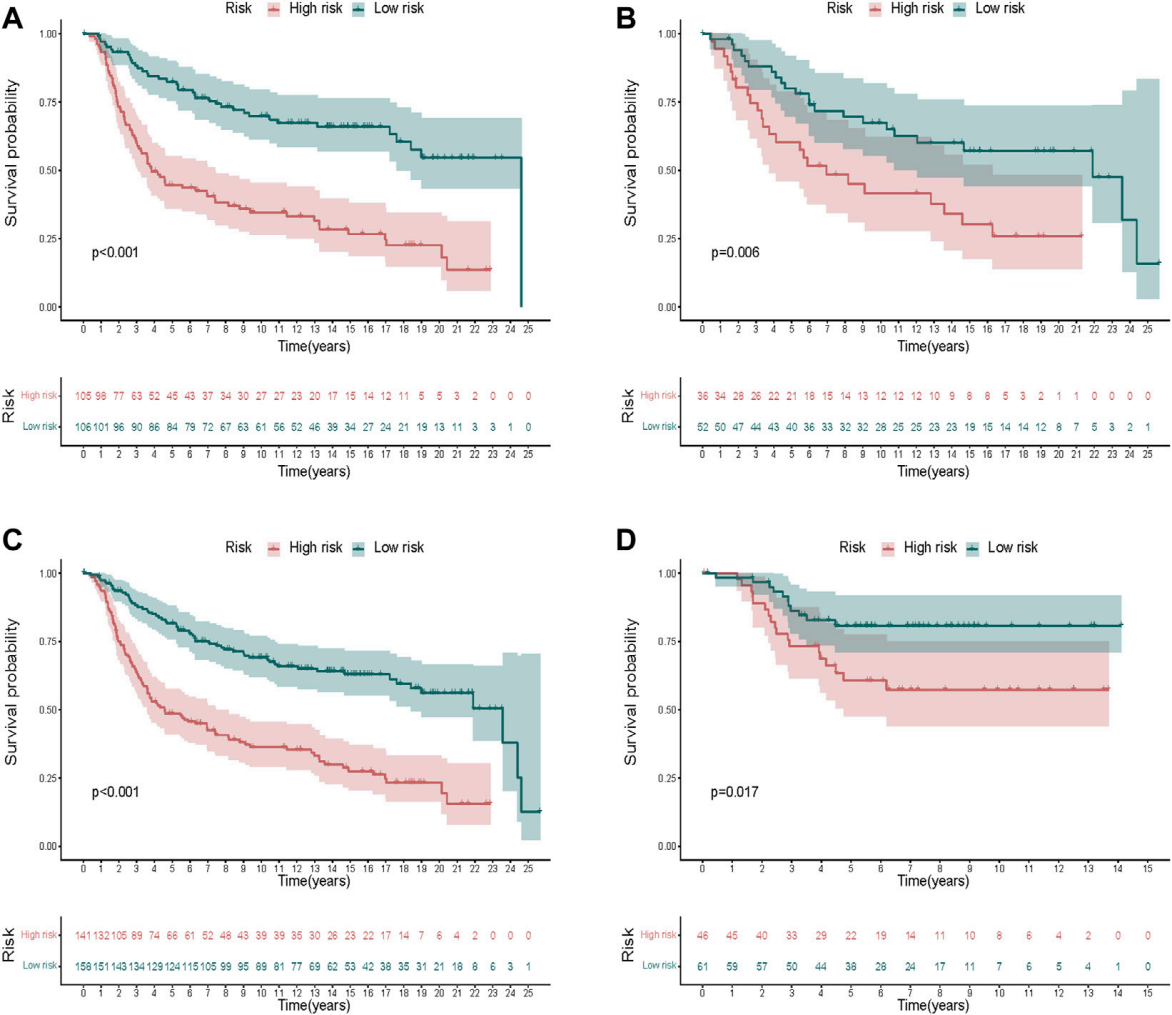
Validation of the risk signature. **(A–D)** KM curves displayed a significantly better OS of the low-risk group in the training cohort **(A)**, the test cohort **(B)**, the whole METABRIC cohort **(C)**, and the GEO cohort **(D)**.

The KM curves exhibited a significantly better OS of the low-risk group in the training cohort (Figure 6A). We also validated the same in the test cohort (Figure 6B), the whole TCGA cohort (Figure 6C), and the GEO cohort (Figure 6D), which showed that the signature could stratify patients into different risks well.

**FIGURE 7:**
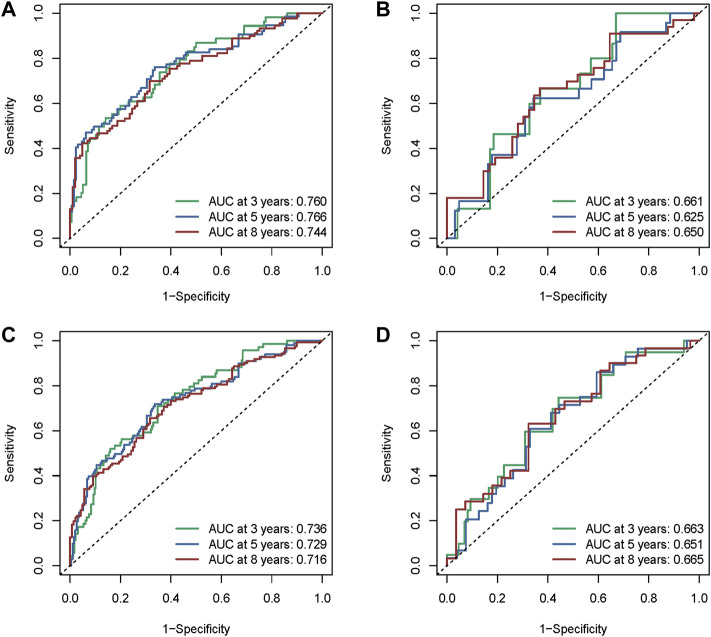
Assessment of the performance of the risk signature. **(A)** The area under the ROC curve (AUC) values were 0.760, 0.766, and 0.744 for the 3-, 5-, 8-year survivals, respectively, in the training cohort. **(B)** AUC values were 0.661, 0.625, and 0.650 for the 3-, 5-, and 8-year survivals, respectively, in the test cohort. **(C)** AUC values were 0.736, 0.729, and 0.716 for the 3-, 5-, and 8-year survivals, respectively, in the whole METABRIC cohort. **(D)** AUC values were 0.663, 0.651, and 0.665 for the 3-, 5-, and 8-year survivals, respectively, in the GEO cohort.

The areas under the ROC curve (AUC) values were 0.760, 0.766, and 0.744 for the 3-, 5-, and 8-year survivals, respectively (Figure 7A), in the training cohort. The AUC values were 0.661, 0.625, and 0.650 for the 3-, 5-, and 8- survivals, respectively, in the test cohort (Figure 7B); those in the whole METABRIC cohort (Figure 7C) were 0.736, 0.729, and 0.716 for the 3-, 5-, and 8-year survivals, respectively; and those in in the GEO cohort (Figure 7D) were 0.663, 0.651, and 0.665 for the 3-, 5-, and 8- survivals, respectively, which demonstrated that the efficacy of the signature was notable.

**FIGURE 8:**
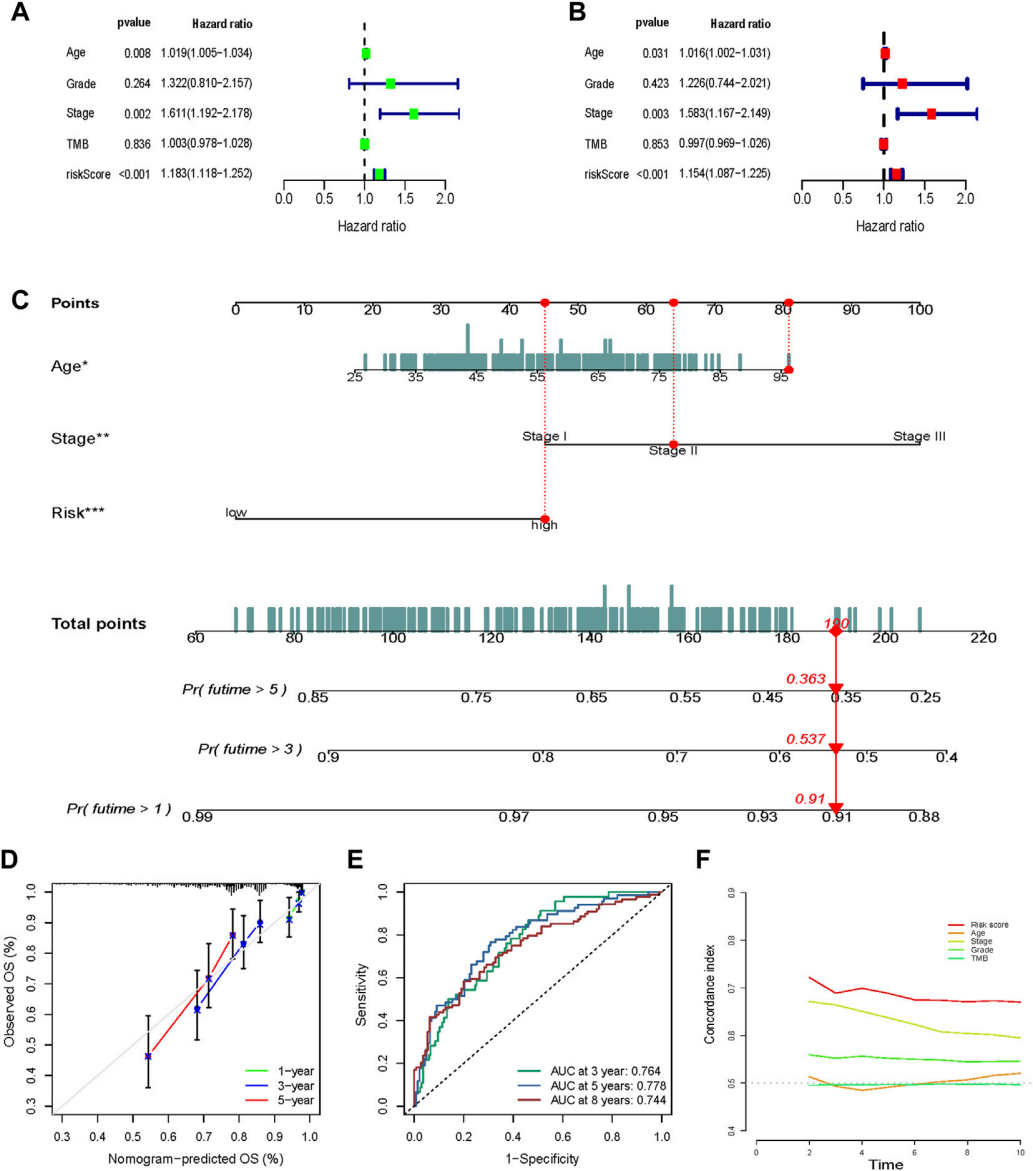
Nomogram construction and validation. Univariate **(A)** and multivariate **(B)** Cox regression analysis indicated that age, stage, and risk score were independent prognostic factors in the METABRIC cohort. **(C)** Age, stage, and risk score were included to construct a nomogram . The points of age, stage, and risk score were calculated with reference to the nomogram, and the total points could facilitate the prediction of prognosis. **(D)** A calibration curve indicated a prominent consistency between the actual observed OS and the predicted OS. **(E)** The efficacy of the nomogram was also assessed using the ROC curves and the AUCs were 0.764, 0.778, and 0.744 for the 3-, 5-, and 8-year survivals, respectively. **(F)** The nomogram exhibited an advantage in C-index versus other clinical traits in prognosis prediction.

Both univariate (Figure 8A) and multivariate (Figure 8B) Cox regression analyses indicated that age, stage, and risk score were independent prognostic factors in the METABRIC cohort.

### Nomogram Construction and Validation

Age, stage, and risk score were included to construct a nomogram to improve the efficacy and facilitate the clinical application of the signature (Figure 8C). The points of age, stage, and risk score were calculated with reference to the nomogram, and the total points could facilitate the prognosis prediction. A calibration curve indicated a prominent consistency between the actual observed and predicted OS (Figure 8D).

The efficacy of the nomogram was also assessed using ROC curves, and the AUC values were 0.764, 0.778, and 0.744 for the 3-, 5-, and 8-year survivals, respectively (Figure 8E). The nomogram showed an advantage in the C-index versus other clinical traits in prognosis prediction (Figure 8F).

### Gene Expression in Particular Cell Types

**FIGURE 9:**
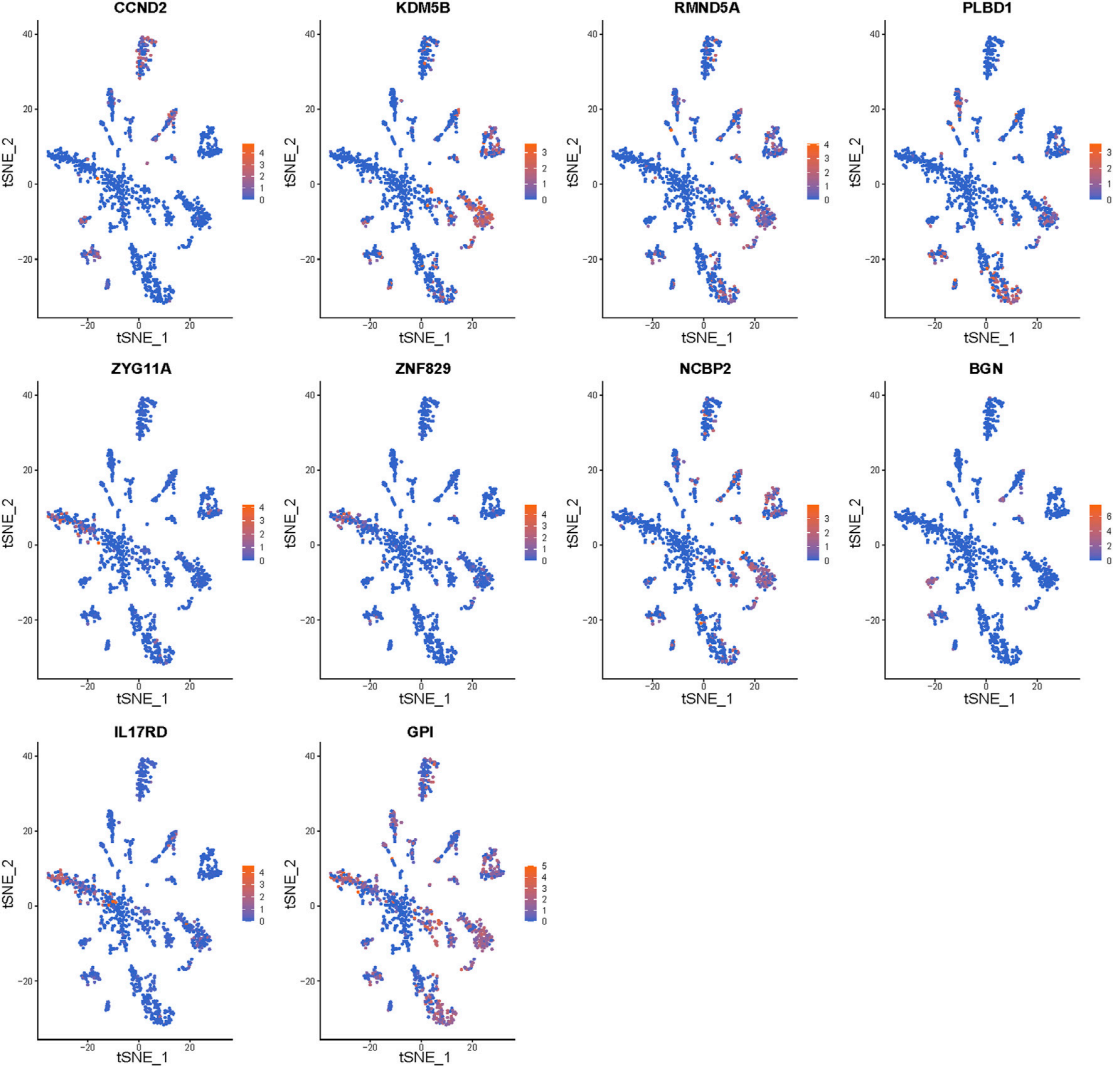
Gene expression in particular cell types. The expression of the 10 signature genes in the cellular landscape.

We further investigated the cell populations in which the 10 genes were expressed (Figure 9). CCND2 was highly expressed in T cells. KDM5B, RMND5A, PLBD1, and NCBP2 were highly expressed in mesenchymal stem cells and epithelial cells. ZYG11A and ZNF829 were highly expressed in epithelial cells. BGN was highly expressed in chondrocytes and fibroblasts, and IL17RD was highly expressed in epithelial cells and tissue stem cells. Finally, GPI was highly expressed in multiple cell types.

### GSVA Differences Between Different Groups

**FIGURE 10:**
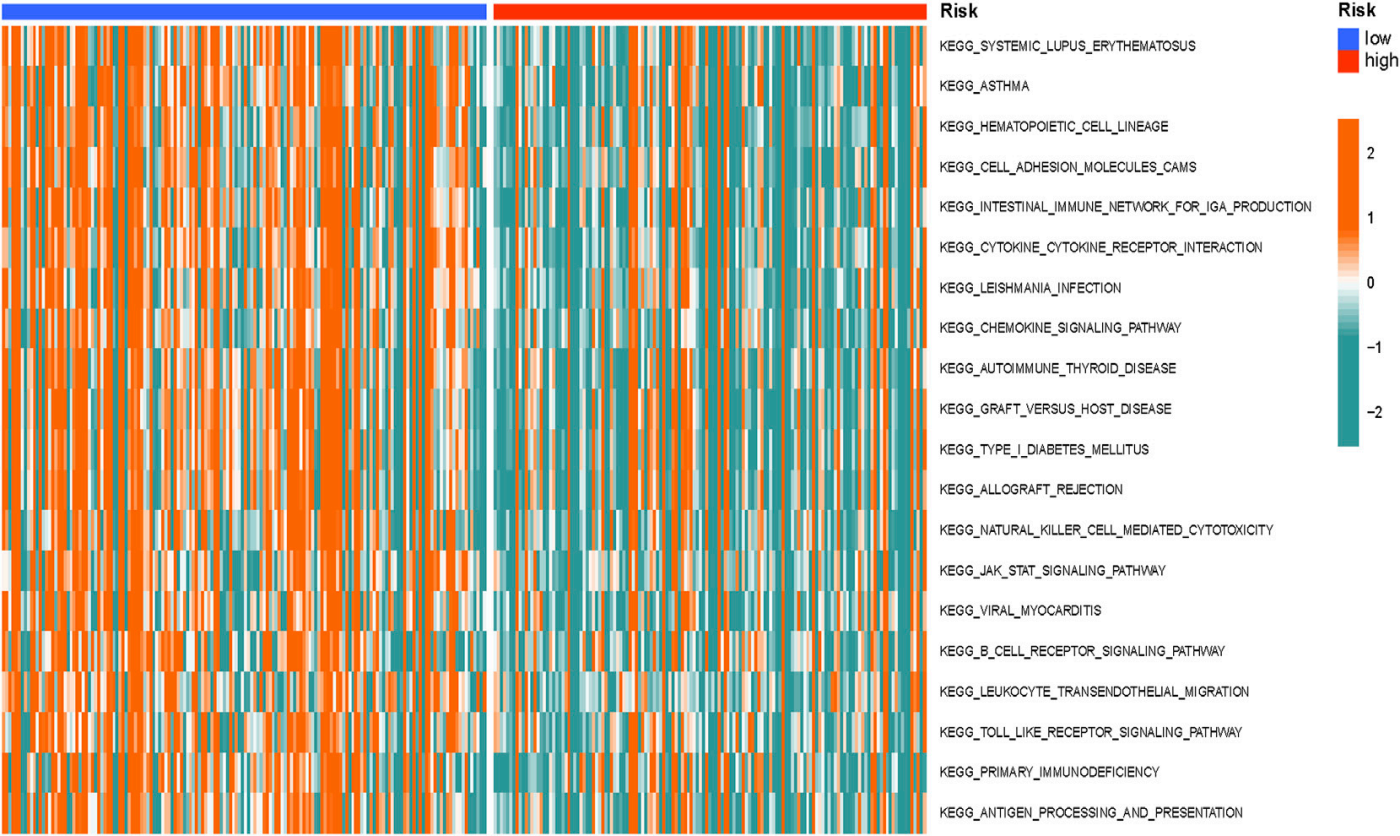
Gene set variation analysis (GSVA) differences between the different groups. The GSVA differences indicated that high-risk groups harbored lower immune-related pathways.

GSVA was conducted to investigate the differentially enriched pathways between different risk groups, and it revealed that high-risk groups harbored lower immune-related pathways such as graft versus host disease and antigen processing and presentation (Figure 10).

### Immune Infiltration Differences Between Different Groups

**FIGURE 11:**
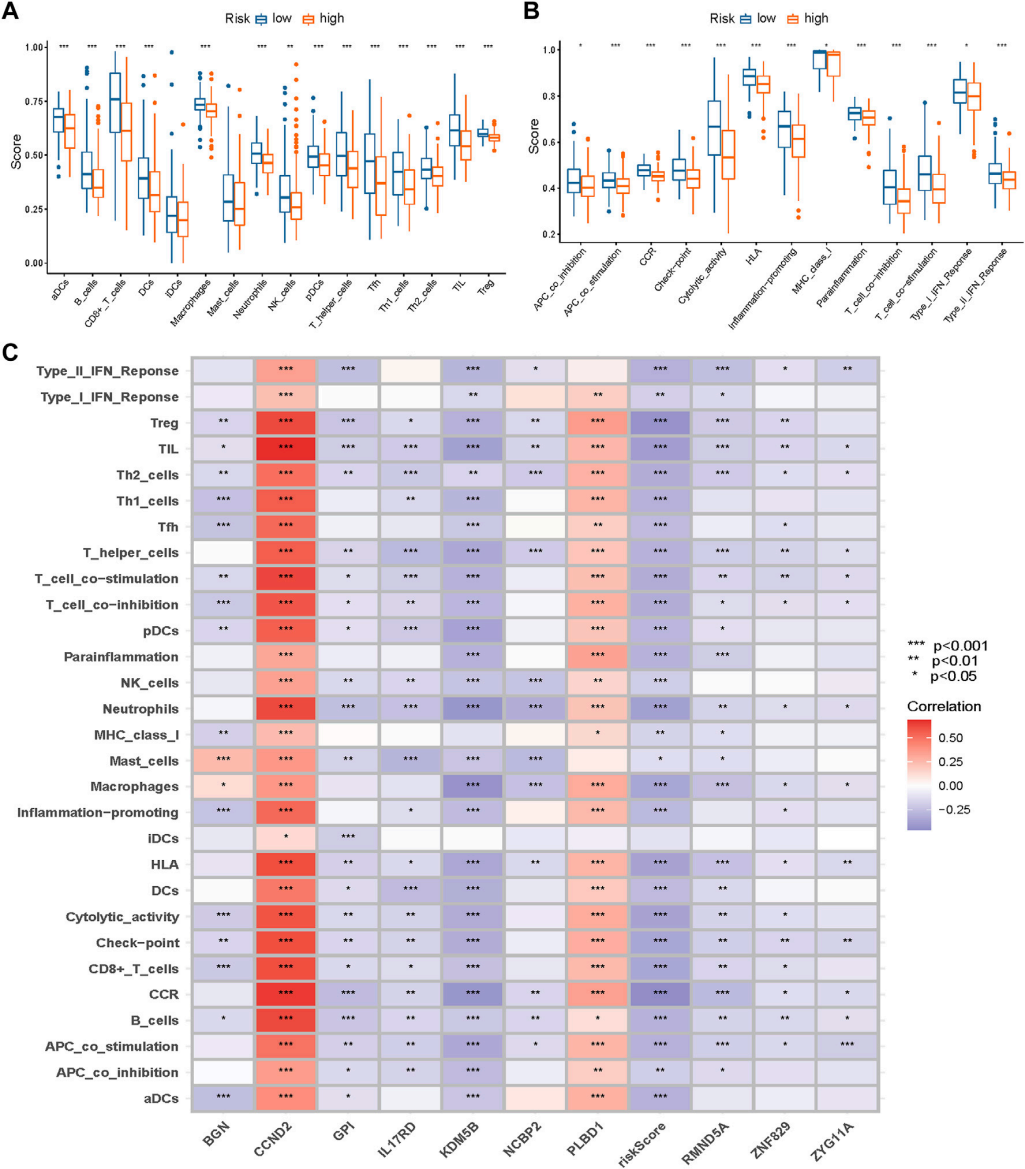
Immune infiltration differences between different groups. Nearly all immune cells **(A)** and activities **(B)** were higher in the low-risk group. **(C)** The correlations between the risk score and genes in the signature and immune infiltration (* *p* < 0.05, ** *p* < 0.01, *** *p* < 0.001).

Considering the differences in immune-related pathways through GSVA, we also investigated the immune infiltration differences between different risk groups. Immune infiltration scores of every sample in the METABRIC dataset are listed in Supplementary Table S3. Nearly all immune cells (Figure 11A) and activities (Figure 11B) were higher in the low-risk group.

The correlations among the risk scores, genes in the signature, and immune infiltration are presented in Figure 11C.

**FIGURE 12:**
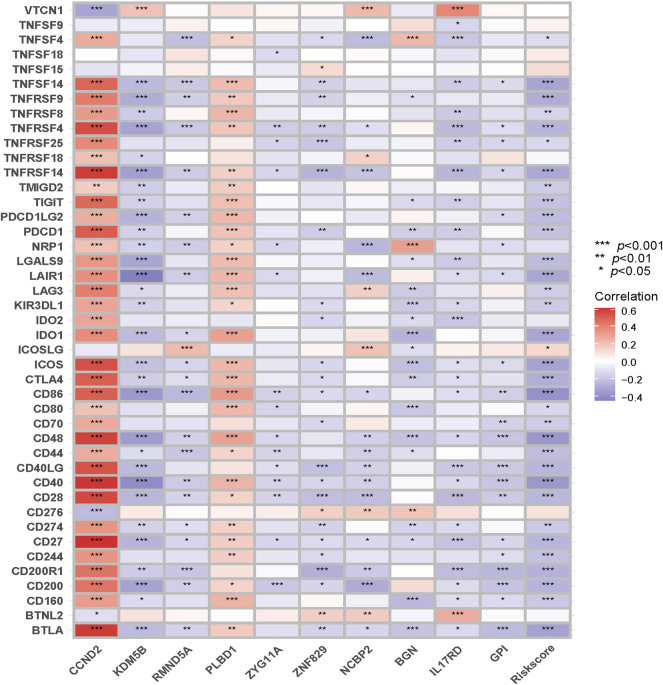
Correlations between the risk score and genes in the signature and immune checkpoint genes.

As the close correlation between the signature and immune infiltration, we investigated the correlations between the risk score, genes in the signature, and immune checkpoint genes, and they are displayed in Figure 12. CCND2 and PLBD1 generally exhibited positive correlations, whereas other genes and risk scores showed the opposite.

## Discussion

TNBC, as an aggressive subtype of breast cancer, has been frequently associated with high rates of mortality. As it is difficult for clinicians to identify the patients who are more vulnerable to therapy failure or disease progression, novel methods are warranted. With the advancements in technology, the NGS technology has been increasingly applied in routine clinical practice (Kamps et al., 2017). Furthermore, the scRNA-seq technology provides precise insight into the cell-specific gene expression and interplay among cells (Packer and Trapnell, 2018). Based on these technologies, we identified DEGs among trajectories that indicated genes related to cell differentiation and further investigated gene-related grade and TMB that might indicate the progression of malignancy. We also developed a signature and demonstrated its efficacy. The signature proved to be an excellent tool to predict the prognosis of TNBC patients in two independent cohorts. We also constructed a nomogram that contained age, stage, and risk to facilitate the clinical practice. The nomogram was more prominent than the signature, as determined through the ROC analysis.

Pseudotime analysis arranges cells based on expression patterns and orders cells along with trajectories, which provides a quantitative measure of cell-type differentiation (Saelens et al., 2019). In this study, through pseudotime analysis of different cells, we endeavored to identify the crucial genes in cell differentiation. Then by WGCNA, we obtained histological grade- and TMB-related genes. The histological grade is closely related to tumor cell atypia and degree of malignancy (Komaki et al., 2006). TMB could indicate the genomic instability (Halbert and Einstein, 2021) and prognosis of breast cancer patients (Thomas et al., 2018). Conjoint analysis might provide clues for the identification of genes that play cardinal roles in tumor initiation and progression.

GSVA is a method used to detect subtle pathway changes over samples through an unsupervised method (Hänzelmann et al., 2013). As the GSVA results indicated immune differences between different risk groups, we investigated the correlations between the risk score and immune infiltration. Interestingly, the results mostly indicate a close correlation between the signature and immune infiltration. The signature composed of genes that might play important roles in tumor progression also exhibited immune infiltration. These findings suggest the essential roles of immune cells in tumor progression and the interplay with tumor cells, which warrant an in-depth understanding. Moreover, all of the above findings might contribute to the notion of cancer immunoediting. Breast cancer has traditionally been considered less immunogenic except TNBC, which is regarded as the optimum subtype for immunotherapy because of high mutational frequency and increased concentrations of tumor-infiltrating lymphocytes (Agostinetto et al., 2022). Our study also showed that immune cells and related activities were lower in the high-risk group. It is reasonable to assume that the high-risk group is relatively more subjected to immune escape.

In our signature, some genes contribute proportionally more to the risk score and deserve further attention and analysis. IL-17RD restrains the motility and invasion of cancer cells and functions as a tumor suppressor (He et al., 2016). IL-17RD mRNA is downregulated in breast cancer, which is significantly correlated with tumor progression (Zisman-Rozen et al., 2007). The ZYG11A expression is negatively correlated with the epithelial ovarian cancer histological grade, which suggests its potential as a candidate tumor suppressor (Achlaug et al., 2021). KDM5B is upregulated in breast cancer and is positively correlated with metastasis (Zhang et al., 2019). However, some genes in previous studies are inconsistent with our study; for instance, upregulated lncRNA GACAT3 in breast cancer enhances its endogenous target CCND2 through sponging miR-497, which was found to be correlated with a poor prognosis (Zhong et al., 2018). This suggests that many genes might play complex roles in different cancer subtypes, contexts, and even different stages of a particular patient’s treatment (Ben-David and Amon, 2020). Still, many genes in our signature are understudied, such as RMND5A and ZNF829. As our studies indicate their roles as oncogenes and contribute considerably to the increase in risk score, they deserve further research.

In summary, during multistep tumorigenesis and progression to higher pathological grades, the genes in the signature might play cardinal roles. Furthermore, when viewed from an immune perspective, the risk score indicates a close correlation with immune infiltration. We believe that our study will facilitate an in-depth understanding of the mechanism of tumor initiation, progression, and immunogenicity and confer an advantage in the rational designing of targeted therapies. In the future, multiomics studies and experiments should be conducted based on the results.

## Conclusion

Cell differentiation and grade- and TMB-related genes were identified using single-cell and bulk RNA-seq data. A 10-gene signature for prognosis prediction in TNBC patients was constructed, and its performance was found to be excellent. Interestingly, the signature was also revealed to be closely related to tumor immune infiltration, which might suggest the crucial roles of immune cells in malignant initiation and progression of TNBC.

## Data Availability

The original contributions presented in the study are included in the article/Supplementary Material, and further inquiries can be directed to the corresponding author.
