# Supplementary figures and images for: Prognosis Prediction Through an Integrated Analysis of Single-Cell and Bulk RNA-Sequencing Data in Triple-Negative Breast Cancer
Source: Front Genet. 2022 Jul 1;13:928175. doi: 10.3389/fgene.2022.928175 (PMC9283578; doi:10.3389/fgene.2022.928175)

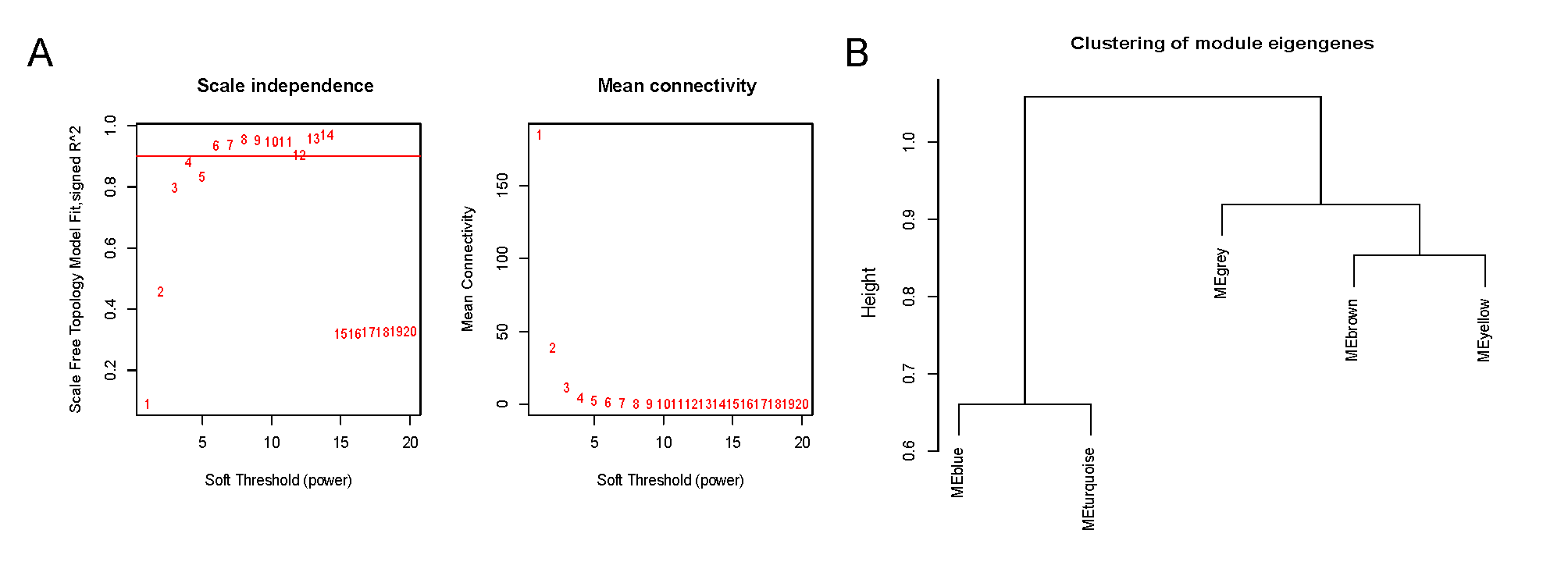

Supplement: Supplementary file 3 [file Image3.TIF]

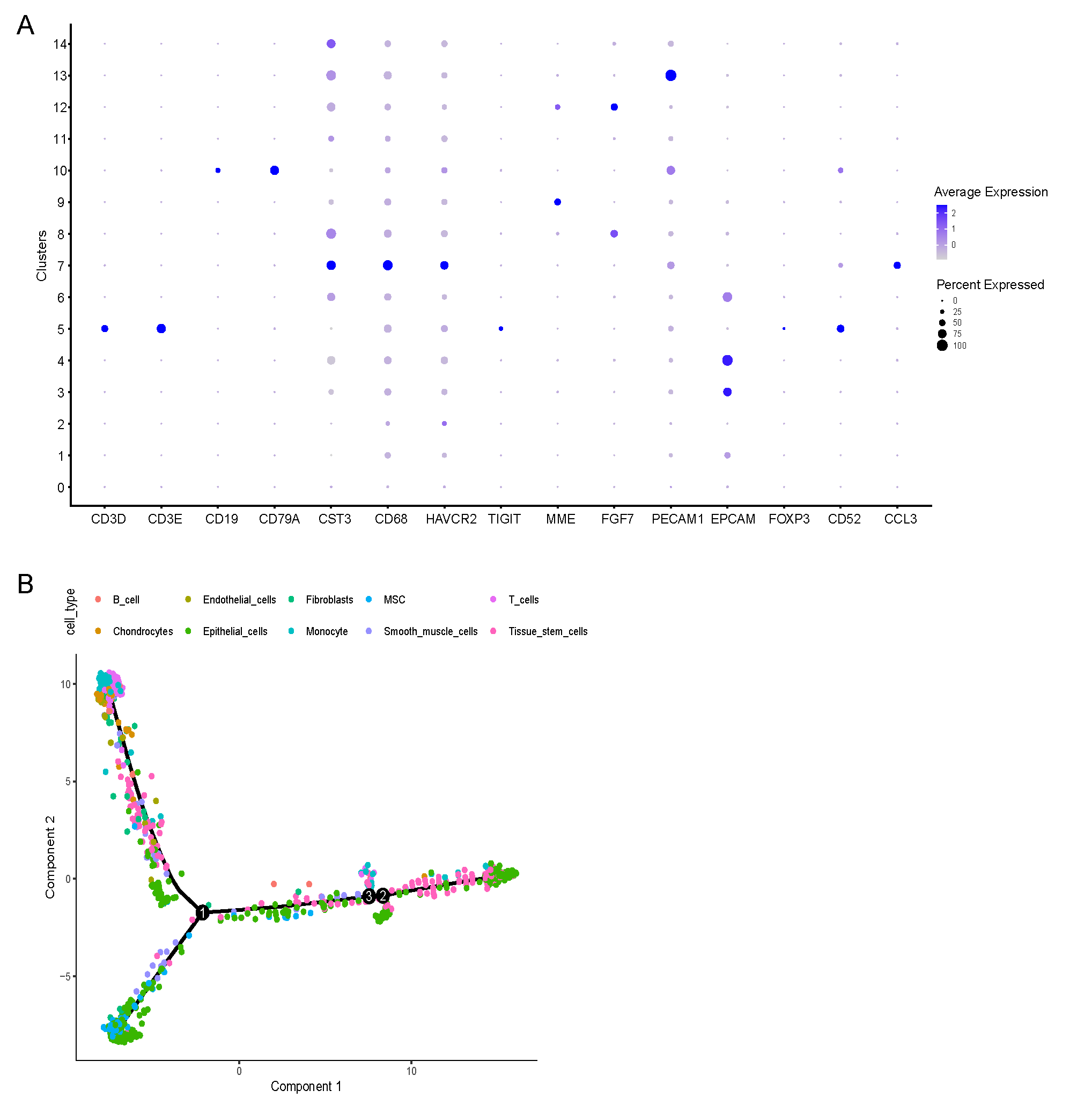

Supplement: Supplementary file 4 [file Image2.TIF]

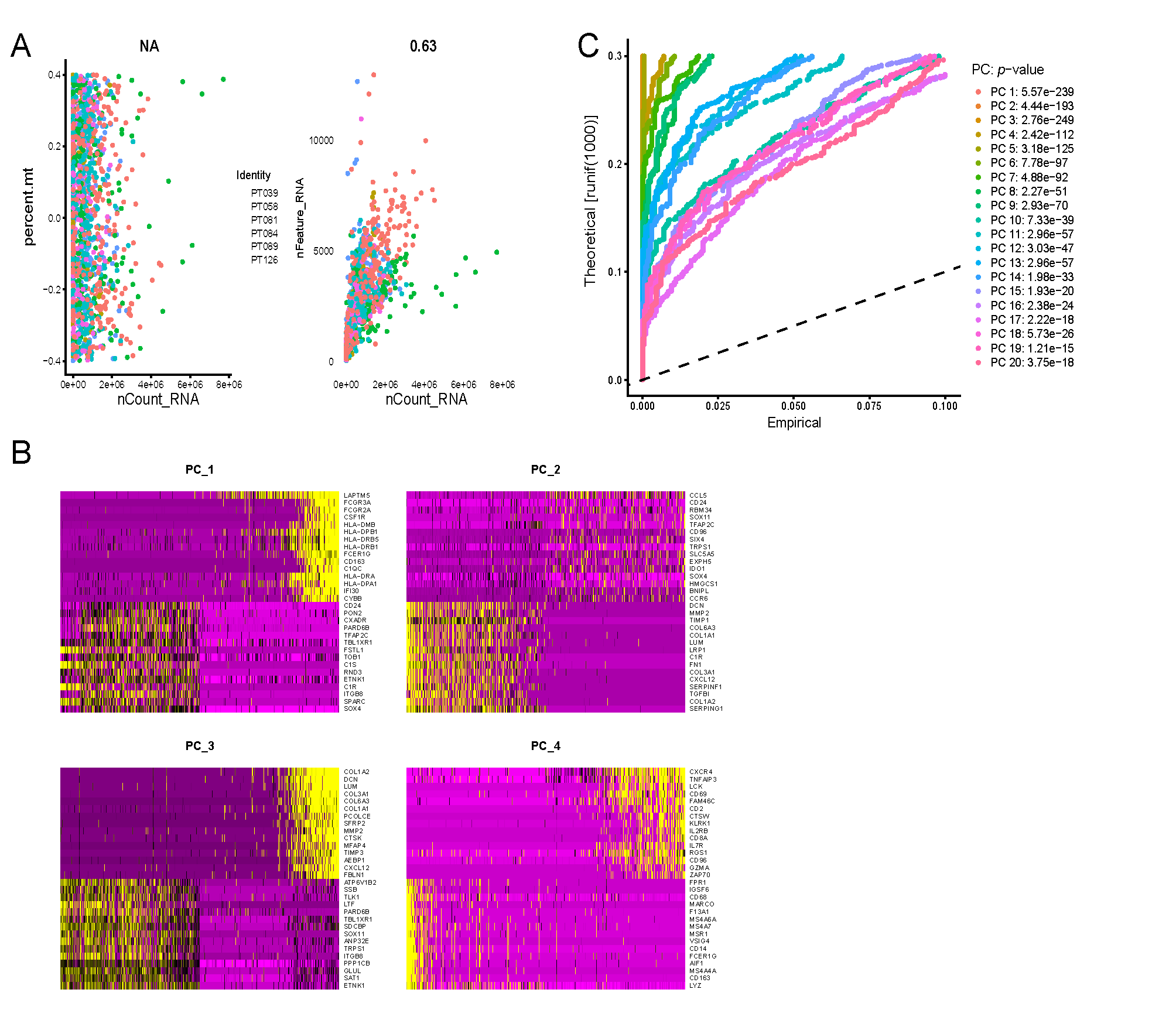

Supplement: Supplementary file 5 [file Image1.TIF]
